# Molecular markers in transitional cell carcinoma of the bladder: New insights into mechanisms and prognosis

**DOI:** 10.4103/0970-1591.38606

**Published:** 2008

**Authors:** Behfar Ehdaie, Dan Theodorescu

**Affiliations:** Department of Urology, University of Virginia, Charlottesville, VA, USA; 1 Department of Molecular Physiology and Biological Physics, University of Virginia, Charlottesville, VA, USA; Mellon Prostate Cancer Institute, University of Virginia, Charlottesville, VA, USA

**Keywords:** Angiogenesis, biomarkers, bladder cancer, carcinogenesis, cell cycle regulators, loss of heterozygosity, molecular markers, prognosis, urothelial carcinoma

## Abstract

Urothelial carcinoma is potentially life-threatening and expensive to treat since for many patients, the diagnosis entails a lifetime of surveillance to detect recurrent disease. Advancements in technology have provided an understanding of the molecular mechanisms of carcinogenesis and defined distinct pathways in tumorigenesis and progression. At the molecular level, urothelial carcinoma is being seen as a disease with distinct pathways of carcinogenesis and progression and thus markers of these processes should be used as both diagnostics and predictors of progression and patient outcome. Herein we present a selective overview of the molecular underpinning of urothelial carcinogenesis and progression and discuss the potential for proteins involved in these processes to serve as biomarkers. The discovery of biomarkers has enabled the elucidation of targets for novel therapeutic agents to disrupt the deregulation underlying the development and progression of urothelial carcinogenesis.

## NATURAL HISTORY

Bladder cancer is a common malignancy. Worldwide, it is the seventh most prevalent cancer, accounting for 3.2% of all malignancies.[1] The highest incidence is seen in industrialized countries and geographic areas where infection with *Schistosoma haematobium* is endemic.[2] In the latter case, the tumor is histologically a squamous cell carcinoma while the majority of the others are of urothelial (transitional) histology. Urothelial carcinoma is the focus of this review.

The majority of patients present with papillary non-muscle-invasive (clinical Stage Ta, T1) urothelial cancer.[3] The natural history of these tumors is significant for local recurrences and relatively infrequent progression to muscle invasion or metastasis.[4] In contrast, a significant number of patients initially presenting with tumors invading the detrusor muscle have coexistent or develop metastasis during follow-up.[4] These tumors are thought to arise *de novo* or evolve from high-grade carcinoma *in situ* (Tis) and despite radical cystectomy and adjuvant chemotherapy, approximately 50% of patients die within five years of diagnosis.[5] Given these findings a theory that urothelial carcinomas develop from two distinct oncogenic pathways has been proposed.[6–9]

Completion of the Human Genome Project has accelerated the pace at which investigators have identified prognostic molecular markers and elucidated the biochemical signaling pathways in the progression of urothelial carcinoma.[10] Molecular and cytogenetic data support the clinical impressions outlined above that low-grade tumors and high-grade tumors may represent distinct signaling pathways resulting in the observed clinical behavior.[6–9] The first may be characterized by low-grade well-differentiated papillary tumors that infrequently invade the detrusor muscle. The second pathway is characterized by carcinoma *in situ* and poorly differentiated tumors, including Grade 3 non-muscle-invasive urothelial cancer, with frequent recurrences and progression to detrusor muscle invasion.

At present, the management of urothelial carcinoma is determined by several tumor-specific factors as well as the patient's overall health status. The treatment of patients with non-muscle-invasive papillary tumors includes transurethral resection (TUR) with or without intravesical chemotherapy or immunotherapy.[11] Standard treatment for patients with muscle-invasive disease (stage ≥ T2) are cystectomy with or without neoadjuvant chemotherapy or bladder-sparing protocols consisting of chemo-irradiation.[12] One of the most clinically challenging tumor presentations is the high-grade non-muscle-invasive Stage T1 tumor. This tumor has already demonstrated the propensity for invasion of the lamina propria, yet because it is not muscle-invasive, it is most often treated with endoscopic therapies such as TUR followed by adjuvant immunotherapy.[13] Unfortunately, a significant number of these patients recur with muscle-invasive disease and require radical treatment. Biomarkers may enable more accurate predictions of which of these tumors have the propensity to progress to muscle-invasive disease and subsequently individualized staging may improve prediction of treatment benefit. Moreover, the elucidation of molecular pathways in tumorigenesis and discovery of biomarkers will enable targeted therapeutic agents to prevent deactivation of cell regulatory mechanisms underlying carcinogenesis.[14]

## NON-MUSCLE-INVASIVE PAPILLARY TUMORIGENESIS

Tumor progression is the result of accumulation of genetic alterations involving the clonal expansion of altered cell with growth advantages through sequential multi-step pathways.[15] Molecular and histopathologic studies indicate that urothelial carcinomas present as a heterogeneous group of tumors that may evolve along two pathways with distinct biological behavior and clinical prognosis. One pathway consists of low-grade papillary tumors that arise from normal urothelial hyperplasia and infrequently (10-15%) progress to muscle invasion.[16] At initial diagnosis 75% of patients with urothelial carcinoma of the bladder have non-muscle-invasive papillary tumors and the five-year survival of these tumors approaches 90% with timely treatment using bladder-sparing techniques.

### Chromosomal aberrations

Loss of heterozygosity (LOH) on Chromosome 9 is found in >50% of all bladder tumors and is more prevalent in the low-grade non-invasive papillary tumors [Table 1a, b].[17] In addition, deletions on Chromosome 9 have been demonstrated in urothelial hyperplasia and normal appearing urothelium adjacent to tumor lesions.[18–20] Aberrations of Chromosome 9 appear to distinguish between the two pathways of bladder cancer tumorigenesis; however, Hartmann demonstrated loss of heterozygosity on Chromosome 9 using fluorescence *in situ* hybridization (FISH) analysis in both dysplastic urothelium and in carcinoma *in situ*.[21] Therefore, deletions on Chromosome 9 may set the stage for tumorigenesis and contribute to both pathways of urothelial carcinogenesis by predisposing urothelial cells to a cascade of genetic alterations. A retrospective study applying FISH to tumor specimens demonstrated that polysomy of Chromosome 17 and LOH on Chromosome 9 were independent predictors of tumor recurrence.[22]

**Table 1a:** Low-grade and non-muscle-invasive urothelial tumors

Chromosomal aberrations		
Loss of heterozygosity of Chromosome 9	Prevalence: >50% of all urothelial tumors and increased prevalence in low-grade tumors	Independent predictor of tumor recurrence
Activating growth factors		
FGFR3	Prevalence: 70% of low-grade non-muscle-invasive tumors. 20% of muscle invasive tumors	Expression of activating mutant FGFR3 gene correlates with non-muscle invasive clinical course
HRAS	Oncogene and transducer of receptor of tyrosine kinase	Primarily associated with non-muscle-invasive clinical course

**Table 1b:** High-grade and muscle-invasive urothelial tumors

Cell cycle regulation		
P53 protein	Gatekeeper in cell cycle control. Prevalence: >50% of high-grade muscle-invasive tumors Cyclin-dependent kinase inhibitor. Prevalence: 64% of radical cystectomy specimens	Nuclear accumulation of p53 predictor of tumor recurrence, progression to muscle invasion and mortality
P21 protein	Cyclin-dependent kinase inhibitor. Prevalence: 64% of radical cystectomy specimens	Nuclear accumulation found to be an independent predictor of tumor recurrence and survival when combined with tumor grade, stage, lymph node stage and p53 status
Rb protein	High-grade and muscle-invasive urothelial tumors harbor inactivating mutations of RB gene	Predictor of time to recurrence and overall survival
Cell adhesion/angiogenesis/cell migration		
E-cadherin	Transmembrane glycoprotein involved in calcium-dependent intercellular adhesion	Decreased E-cadherin expression independent predictor of tumor progression to muscle invasion and decreased disease-specific survival
Angiogenesis	Histologically measured by microvessel density. Biochemically measured by serum VEGF	Increased microvessel density independent predictor of disease free and overall survival. Increased serum VEGF independent predictor of poor disease survival on univariate analysis
RhoGD12	Metastatic suppressor gene. GTPases play a central role in coordinating signal transduction pathways that affect actin and tubulin a cytoskeleton and cell migration	Increased protein expression independent prognostic marker of disease relapse following radical cystectomy

### Activating growth factor signals

Low-grade non-muscle-invasive tumors demonstrate constitutive activation of cellular growth factor signaling pathways, including receptor tyrosine kinases and the RAS pathway.[23,24] Fibroblast growth factor receptor 3 (FGFR3) is one of four members of a tyrosine kinase receptor family that play a role in embryonic development, cell growth, differentiation, proliferation and angiogenesis.[25] Approximately, 70% of low-grade non-muscle-invasive papillary tumors have been shown to demonstrate FGFR3 gene mutations compared with 20% of invasive tumors. The expression of activating mutant FGFR3 gene in urothelial cell carcinoma correlates with noninvasive clinical course.[26–31] A study by van Rhijn examined 260 bladder cancer specimens and demonstrated FGFR3 genetic alterations were found predominantly (60%) in low-grade non-muscle-invasive tumors and were associated with favorable outcomes.[32] Additionally, van Rhijn classified tumors based on FGFR3 and MIB-1 status and demonstrated more accurate prognostic information compared to standard clinicopathologic classification.[33] Two studies concluded that the FGFR3 gene status did not correlate with disease progression in high-grade non-muscle-invasive bladder cancer[34,35] and a third study did not find immunohistochemistry measured expression of FGFR3 an independent predictor of recurrence.[36] Together, these studies suggest that in high-grade tumors, FGFR3 is no longer contributing to the maintenance of the malignant phenotype.

The HRAS is a human oncogene and key transducer of the receptor tyrosine kinases. Mutations in HRAS constitutively activate the HRAS protein and enable propagation of the growth factor signal. Mutations in HRAS are primarily associated with non-muscle-invasive urothelial carcinoma and transgenic mouse models have demonstrated evolution of urothelial hyperplasia to low-grade non-invasive papillary tumors.[37,38] Therefore, overexpression of activated HRAS is sufficient to induce urothelial tumorigenesis and the receptor tyrosine kinase – Ras pathway contributes to the low-grade non-invasive papillary pathway of urothelial tumorigenesis.

## HIGH-GRADE UROTHELIAL CARCINOGENESIS

While most tumors invading the detrusor muscle are usually diagnosed in patients with no history of papillary tumors, a significant minority occur in patients with previous high-grade Stage T1 or Tis/carcinoma *in situ* cancers. High-grade urothelial tumors represent 40% of initially diagnosed bladder cancers and more than half of which are muscle-invasive or more extensive at the time of diagnosis.[39] The molecular features of these tumors will be discussed below.

### Cell cycle regulation

The p53 tumor suppressor protein, encoded by the TP53 gene, is a key gatekeeper in cell cycle control.[40] The p53 protein inhibits cell cycle progression at G1 – S transition and plays an integral role in molecular pathways related to carcinogenesis and response to therapy, including cell cycle regulation, angiogenesis, apoptosis and DNA repair.[41] Mutations involved in the p53 protein dysfunction occur in two phases: initially one allele is affected, followed by loss of a second, wild-type allele.[42] Overexpression of p53 as determined by immunohistochemistry is routinely used to measure TP53 mutations which are infrequent in low-grade non-invasive papillary tumors, but very common (>50%) in high-grade invasive urothelial tumors and CIS tumors.[21,43,44] The product of a mutated TP53 gene has been shown to be a metabolically stable protein with a long half-life. In contrast, wild-type p53 protein has a short half-life, measured as only 6 to 30 min and therefore it does not accumulate in high enough levels to be detected by standard immunohistochemical methods. A mututed p53 protein, in contrast, is detectable by immunohistochemistry studies. In most studies, p53 nuclear accumulation is predictive of tumor recurrence, progression and mortality.[45–52] However, a meta-analysis by Malats reviewing 117 studies spanning 10 years of research concluded that the evidence is not yet sufficient to conclude whether changes in P53 act as markers of outcome in patients with bladder cancer.[53] A second review by Schmitz-Drager analyzing 43 trials determined that in only one-half did p53 retain prognostic significance upon multivariate analysis. Furthermore, comparison between trials yielded significant differences in technical aspects of study design, including variable cut-off values for IHC and selection of antibodies.[54] Hence, it would appear that expression of p53 has molecular significance in bladder carcinogenesis and closely correlates with disease progression, but its prognostic utility is confounded by its close association with standard clinical and pathologic prognostic features. Nevertheless, a small independent effect cannot be excluded.

The expression of p21 protein, a cyclin-dependent kinase inhibitor and an important downstream target of p53, is downregulated in the majority of urothelial carcinomas with TP53 mutations. In a retrospective study of radical cystectomy patients with long patient follow up, nuclear accumulation of p21 as detected by immunohistochemistry, was a characteristic of 64% of specimens and was an independent predictor of tumor recurrence and of survival when assessed with grade, tumor stage, lymph node status, and p53 status.[55] In a second study retrospectively examining radical cystectomy specimens, alteration of p21 expression was independently associated with disease progression and disease-specific survival.[56] Recently, a study retrospectively reviewing non-muscle-invasive bladder tumor specimens from transurethral resections (TUR) demonstrated that altered p21 expression was independently associated with disease progression but not recurrence.[57] Perhaps evaluation of p53 and p21 expression have synergistic effects on bladder cancer outcome enabling stratification of patients into different risk groups.

The retinoblastoma gene (RB) encodes a nuclear phosphoprotein (Rb), the phosphorylation of which has been directly involved in epithelial tumorigenesis, regulating development, differentiation, cell cycle restriction and apoptosis.[58] The active, dephosphorylated Rb protein binds and inactivates the transcription factor E2F, thereby inhibiting DNA synthesis.[59] High-grade and invasive urothelial tumors harbor inactivating mutations of the RB gene.[60] In a retrospective study using immunohistochemical analysis of high-grade and invasive bladder cancer specimens, p53, p21 and Rb status were independent predictors of time to recurrence and overall survival.[61] Urothelial tumors with alterations in both p53 and Rb expression had increased rates of recurrence and progression and worse survival then tumors harboring defects of either gene.[62] In addition, studies in transgenic mice with functionally inactivated p53 and Rb proteins develop exclusively high-grade CIS lesions that progress to muscle-invasive disease.[63] Therefore, p53 and RB act as tumor suppressors and may have a synergistic role in preventing evolution of high-grade urothelial tumors.

### Cell adhesion, angiogenesis and migration

Tumor invasion is a key determinant of patient outcome and relies on not only intrinsic genetic factors of the tumor cells, but also on the local environment within which tumorigenesis occurs. The primary features of the pathway for invasive urothelial tumors will be discussed below and include decreased cell-cell adhesion, increased breakdown of extracellular matrix and increased angiogenesis.

E-cadherin is a member of the family of transmembrane glycoproteins involved in calcium-dependent intercellular adhesion. Alteration of E-cadherin expression induces a defect in cell-cell adhesion and is primarily seen in high-grade muscle-invasive tumors.[64] In a study of patients who underwent radical cystectomy, reduced expression of E-cadherin correlates with increased stage and grade of bladder cancer and is an independent predictor for disease progression and lymph node metastasis.[65] In retrospective studies that examined high-grade non-muscle-invasive bladder cancers, decreased E-cadherin expression identified by immunohistochemistry independently predicted tumor progression to invasive disease and decreased disease-specific survival.[66,67]

Angiogenesis is critical for tumor proliferation and invasion in maintaining the supply of oxygen and nutrients. Histologically, micro-vessel density (MVD) is measured to estimate the degree of angiogenesis.[68] A study examining 164 patients with muscle-invasive bladder cancer using immunohistochemistry to determine micro-vessel density demonstrated angiogenesis to be an independent prognostic indicator of disease-free and overall survival when evaluated in the presence of histological grade, pathologic stage and regional lymph node status.[69] The serum levels of vascular endothelial growth factor (VEGF), a pro-angiogenic molecule and predictor of metastatic disease and associated with poor disease-free survival, however, do not demonstrate independent prognostic information on multivariate analysis.[70]

Cell migration is a critical factor of metastasis and contributes to both cancer cell invasion into vasculature and penetration of host tissue at distant sites.[71] The Rho family of GTPases plays a central role in coordinating and regulating the signal-transduction pathways that affect actin and tubulin cytoskeletons and cell migration. The Rho/ROCK pathway has been significantly associated with invasion and metastasis of bladder cancer.[72] Reduced expression of the newly discovered metastasis suppressor gene RhoGDI2 correlates with increased invasive and metastatic activity in bladder carcinogenesis.[73] A study of bladder specimens demonstrated that decreased RhoGDI2 mRNA and protein expression were independent prognostic markers of disease relapse following radical cystectomy.[74]

## CLINICAL APPLICATIONS

Development and discovery of molecular markers of prognosis in bladder cancer is an active area of translational research. The presence of a multitude of molecular markers that reflect bladder cancer progression suggests that multi-panel assays will likely be more predictive than the evaluation of a single marker. These can be combined in currently used clinical nomograms of prognosis if they offer additional predictive information.[75] Furthermore, combined application of array-based genomic and proteomic expression profiling may lead to the discovery of additional prognostic biomarkers involved in tumor progression.[76–78]

However the routine clinical use of incorporating molecular markers in a prognostic setting first requires prospective studies that demonstrate independent prognostic information over standard clinical and histopathologic parameters. The International Consensus Panel on Bladder Tumor Markers recently concluded that based on the current evidence, none of the current prognostic molecular markers are sufficiently validated to be used in the clinical management of patients with urothelial carcinoma of the bladder.[79] Furthermore, given the multiplicity, complexity and crosstalk of the biochemical pathways involved in the tumorigenesis and progression of bladder cancer, a single marker may prove to be inadequate to accurately stratify tumors with similar histopathologic characteristics into distinct prognostic pathways.

Understanding the molecular basis of tumor progression can lead to both identification of biomarkers and targets for therapy. Indeed, some may be both. For example, agents that restore tumor suppressor functions of p53 or Rb are available.[80,81] Two recent reviews discuss new agents using targeted therapeutic regimens.[82,83] Emerging therapies that target specific pathways and cell signaling molecules will need to be evaluated together with conventional therapies including chemotherapy, immunotherapy and radiotherapy.[84]

## CONCLUSION

The past decade has seen an exponential accumulation of studies and information on molecular markers in urothelial carcinoma of the bladder. As the complex molecular mechanisms and biological pathways that lead to urothelial tumorigenesis are increasingly understood, biological markers are being discovered that offer to enhance standard clinicopathologic information and thus optimize predictive clinical tools and personalize the therapeutic approaches to these patients to reduce the risk of progression. However, based on a limited review of current data, it would appear that none of the markers discussed here have demonstrated at this time, sufficient prognostic value to warrant their use in the clinical management of patients with bladder cancer. As this review was not intended to be comprehensive, we apologize to the many authors whose work we were not able to cite herein.

## GLOSSARY OF TERMS

1. Urothelial Carcinoma (Transitional cell carcinoma): a malignant neoplasm derived from transitional epithelium, occurring chiefly in the urinary bladder, ureter or renal pelvis.
2. Loss of Heterozygosity (LOH): In a heterozygote, the loss of one of the two alleles at one or more loci in a cell lineage or cancer cell population due to chromosome loss, deletion or mitotic crossing-over.
3. Fluorescence *in situ* hybridization (FISH): A technique that employs fluorescent molecular tags to detect DNA or RNA probes hybridized to complementary chromosomes or chromatin; useful for genetic mapping and detecting chromosomal abnormalities.
4. Tyrosine kinase: an enzyme that can transfer a phosphate group to a tyrosine residue in a protein; these enzymes are a subgroup of the larger class of protein kinases that function in signal transduction to regulate enzyme activity.
5. RAS pathway: The Ras gene family consists of H-Ras, N-Ras and K-Ras. The Ras proteins are typically small triphosphate-binding proteins and are the common upstream molecule of several signaling pathways that play a key role in signal transduction, cytoskeletal integrity, cellular proliferation, adhesion, apoptosis and migration.
6. Angiogenesis: physiological process involving the formation of new blood vessels from preexisting vessels. This is a normal process in growth and development, as well as in wound healing. However, this is also a fundamental step in the transition of tumors from a dormant state to a malignant state.
7. p53 protein: tumor suppressor protein encoded by the TP53 gene located on Chromosome 17p13.1. It inhibits phase-specific cell cycle progression (G1-S) and regulates its control through the transcriptional activation of p21^WAF1/CIP1^.[85]
8. Gene microarray analysis: collection of microscopic DNA fragments, representing single genes, arrayed on a solid surface by covalent attachment to chemically suitable matrices. Qualitative or quantitative measurements with DNA microarrays utilize the selective nature of DNA-DNA or DNA-RNA hybridizaton under high-stringency conditions and fluorophore-based detection. DNA arrays are commonly used for monitoring expression levels of thousands of genes simultaneously.
